# Dietary Spray-Dried Porcine Plasma Reduces Neuropathological Alzheimer’s Disease Hallmarks in SAMP8 Mice

**DOI:** 10.3390/nu13072369

**Published:** 2021-07-10

**Authors:** Cristina Rosell-Cardona, Christian Griñan-Ferré, Anna Pérez-Bosque, Javier Polo, Mercè Pallàs, Concepció Amat, Miquel Moretó, Lluïsa Miró

**Affiliations:** 1 Department of Biochemistry and Physiology, Faculty of Pharmacy and Food Sciences, Institute for Nutrition and Food Safety, Universitat de Barcelona (UB), 08028 Barcelona, Spain; cristina.rosell@ub.edu (C.R.-C.); anna.perez@ub.edu (A.P.-B.); camat@ub.edu (C.A.); mmoreto@ub.edu (M.M.); 2 Department of Pharmacology, Toxicology, and Medicinal Chemistry, Faculty of Pharmacy and Food Sciences, Institute of Neurosciences, CIBERNED, Universitat de Barcelona (UB), 08028 Barcelona, Spain; christian.grinan@ub.edu (C.G.-F.); pallas@ub.edu (M.P.); 3 APC Europe S.L.U., 08403 Granollers, Spain; javier.polo@apc-europe.com

**Keywords:** neuroinflammation, dietary supplementation, aging, plasma proteins, Alzheimer’s disease, SAMP8

## Abstract

Alzheimer’s disease (AD) is characterized by the aberrant processing of amyloid precursor protein (APP) and the accumulation of hyperphosphorylated tau, both of which are accompanied by neuroinflammation. Dietary supplementation with spray-dried porcine plasma (SDP) has anti-inflammatory effects in inflammation models. We investigated whether dietary supplementation with SDP prevents the neuropathological features of AD. The experiments were performed in 2- and 6-month-old SAMP8 mice fed a control diet, or a diet supplemented with 8% SDP, for 4 months. AD brain molecular markers were determined by Western blot and real-time PCR. Senescent mice showed reduced levels of p-GSK3β (Ser9) and an increase in p-CDK5, p-tau (Ser396), sAPPβ, and the concentration of Aβ_40,_ (all *p* < 0.05). SDP prevented these effects of aging and reduced *Bace1* levels (all *p* < 0.05). Senescence increased the expression of *Mme1* and *Ide1* and pro-inflammatory cytokines (*Il-17* and *Il-18*; all *p* < 0.05); these changes were prevented by SDP supplementation. Moreover, SDP increased *Tgf-β* expression (*p* < 0.05). Furthermore, in aged mice, the gene expression levels of the microglial activation markers *Trem2*, *Ym1*, and *Arg1* were increased, and SDP prevented these increases (all *p* < 0.05). Thus, dietary SDP might delay AD onset by reducing its hallmarks in senescent mice.

## 1. Introduction

The prevalence of Alzheimer’s disease (AD), already the most common type of dementia, is increasing at an alarming rate due to the life expectancy improvements of recent decades [1]. This neurodegenerative disorder, which leads to cognitive decline, is characterized by aberrant amyloid precursor protein (APP) processing, the deposition of β-amyloid (Aβ) peptides such as Aβ_40_ and Aβ_42_, and the aggregation of hyperphosphorylated tau (p-tau) protein, all processes that are accompanied by neuronal loss [2]. Aβ peptides tend to self-aggregate as oligomers in β-amyloid or senile plaques, with extracellular Aβ accumulation caused by cleavage of APP by the β-secretase BACE1 [3].

Tau proteins are microtubule-associated proteins that stabilize microtubules in neuronal axons. Hyperphosphorylation of tau (i.e., p-tau) leads to the accumulation of neurofibrillary tangles (NFTs) and toxic species of soluble tau, which disrupt normal synaptic and neuronal function [4]. The kinases responsible for tau phosphorylation are glycogen synthase kinase 3β (GSK3β) and cyclin-dependent kinase 5 (CDK5). GSK3β, in addition to its role in the pathogenesis of NFTs, also promotes pro-inflammatory pathways through nuclear factor kappa B (NF-κB) activation. Indeed, it is inhibited when phosphorylated at Ser9, which stimulates its glucose synthesis-related physiological effects [5]. Under pathological conditions, CDK5 becomes hyperactive and causes aberrant hyperphosphorylation of tau proteins in NFTs [6] and interferes with the proteolytic processing of APP, modulating Aβ by affecting the secretases, which are critical for the hydrolysis of APP [7]. Therefore, these enzymes play major roles in AD by affecting amyloid processing and NFTs formation.

Another of the main features of AD pathogenesis is inflammation. During senescence, there is an increase in the low-intensity inflammatory state known as inflammaging [8]. The role of neuroinflammation in AD has been determined using genome-wide association studies that identified more than 20 gene variants as risk factors for developing AD [9]. For instance, p-tau can activate the MAPK pathway and the NF-κB transcription factor pathway, which in turn increase IL-6 levels [10]. Similarly, Aβ can activate microglia, increasing the expression of cytokines [11]. Microglial cells are the immune system of the central nervous system: they can act as phagocytes, removing Aβ, and they play an important role in neuronal homeostasis and synaptic plasticity [12]. Indeed, activated microglia represent a noteworthy feature in AD [13].

Dietary supplementation with spray-dried porcine plasma (SDP) is widely used in farming as an alternative to antibiotics [14]. SDP promotes growth performance, feed efficiency, and animal survival [15,16]. Furthermore, SDP exerts anti-inflammatory effects in different models of inflammation, such as in the intestinal [17,18,19,20], pulmonary [21], and genitourinary mucosae in a mouse model, inducing low pregnancy rates by stress [22]. It also stimulates the growth of probiotic species in the gut microbiota and the expression of mucosal regulators of anti-inflammatory pathways [23]. Moreover, SDP supplementation prevents cognitive decline in senescent mice [24].

This study aimed to determine whether SDP supplementation could exert a neuroprotective effect against AD hallmarks in SAMP8 mice, a model of age-related cognitive decline and AD. These mice present several neuropathological features of AD, such as aberrant APP processing [25], oxidative stress damage [26], and neuroinflammation [27,28,29]. Thus, we used this rodent model to study some of the most important molecular mechanisms associated with AD.

## 2. Materials and Methods

### 2.1. Animals, Experimental Design and Diets

Experiments were conducted using mice prone to senescence (SAMP8 strain) obtained from Envigo (Bresso, Italy). The colony generated was kept at the animal facility of the Faculty of Pharmacy and Food Sciences of the Universitat de Barcelona under stable temperature and humidity conditions, with a 12 h:12 h light/dark cycle. All animal experiments were performed following the Guide for the Care and Use of Laboratory Animals, and the protocols used in this study were approved by the Ethics Committee for Animal Experimentation of the Universitat de Barcelona and the Catalan government (ref. 484/16 and 9272, respectively). Animals were housed individually and fed experimental diets (Control or SDP) for 4 months.

SDP is a protein-rich ingredient obtained from industrial fractionation of blood from healthy pigs. Once the cellular fraction is separated, plasma is then concentrated and spray-dried, thus obtaining the so-called “spray-dried plasma” (SDP). During this process, plasma proteins are exposed to high temperatures for a very short period, and this has the advantage over conventional drying in that the proteins are not denaturalised and preserve their biological activity [30]. The SDP is considered a safe product, and its manufacturing process includes several biosafety steps [31]. Diets were balanced for energy and total N, and lysine and methionine were formulated to meet the National Research Council requirements [32] for laboratory animals. The composition of the experimental diets has been published previously [24] and is detailed in Table 1.

The experimental design of this study included 3 groups of 6–8 animals/group: 2M, 2-month-old mice fed commercial standard feed; 6M-CTL, 6-month-old mice fed with control diet for 4 months; and 6M-SDP, 6-month-old mice fed with SDP diet for 4 months.

### 2.2. Sample Collection

At the end of the experimental period, mice were anaesthetized with ketamine: xylazine (100 mg/kg:10mg/kg); blood was obtained by cardiac puncture and the animal was euthanized during the experimental intervention. The brain was collected, quickly frozen in liquid nitrogen, and stored at −80 °C until further analysis.

### 2.3. Sample Homogenization

Samples of brain tissue were homogenized using a Polytron (PRO Scientific Inc, Oxford, CT, USA) at 20,000 rpm in a lysis buffer containing 50 mM Tris-HCl, 150 mM NaCl, 2 mM EDTA, 1% Triton, 1 mM PMSF, 1 mM DTT, and 2% (*v/v*) inhibitor protease cocktail. The homogenate was centrifuged at 4 °C at 955× *g* for 20 min. All reagents were provided by Sigma-Aldrich (St. Louis, MI, USA).

### 2.4. Western Blot

Western blot procedure was performed, as described previously by Moretó et al. [23]. Briefly, samples of homogenized brain tissue were quantified for protein concentration using the Bradford method (Bio-Rad, Munich, Germany). Equal amounts of protein (50 μg) were separated on 5–18% SDS-PAGE and transferred to polyvinylidene difluoride membranes (Bio-Rad, Munich, Germany). Membranes were blocked by incubation for 90 min at room temperature in Tris-buffered saline containing 0.1% Tween 20 (TBST) and 5% dry milk. Afterwards, membranes were incubated overnight at 4 °C with primary antibodies: β-amyloid (1/500, Abcam, Cambridge, UK); tau (1/1000, Millipore, Billerica, MA, USA); p-tau (Ser396, 1/1000, Invitrogen, Carlsbad, CA, USA); CDK5 (1/1000, Santa Cruz Biotech, Dallas, TX, USA); p-CDK5 (1/1000, Abcam, Cambridge, UK); GSK3 (1/1000, Cell Signaling, Danvers, MA, USA); p-GSK3 (Ser9, Abcam, Cambridge, UK); sAPPα (1/1000, Covance, Princeton, NJ, USA); sAPPβ (1/1000, Covance, Princeton, NJ, USA); β-actin (1/30000, Sigma Aldrich, St. Louis, MI, USA). Membranes were washed and incubated with HRP-conjugated secondary antibodies (Sigma-Aldrich, St. Louis, MI, USA) for 2 h at room temperature. Protein bands were visualized using a chemiluminescence detection kit Clarity and ChemiDoc XRS+ instrument (both from Bio-Rad, Munich, Germany). After their detection, hybridization bands were quantified using the plug-in Gel Lanes fit from ImageJ software.

### 2.5. ELISA Assay

Cortical tissues were homogenized in cold 5 M guanidine-HCl/50 mM Tris buffer containing protease inhibitor cocktail (Sigma Aldrich, St. Louis, MI, USA). The quantification of amyloid-β_40_ and amyloid-β_42_ was performed with the mouse Aβ_40_ and Aβ_42_ ELISA Kits (both from Invitrogen, Carlsbad, CA, USA), following manufacturer’s instructions.

### 2.6. Real-Time PCR

RNA extraction of the cortex and reverse transcription were carried out as described previously [24]. The primers used are shown in Table 2. Product fidelity was confirmed by melt curve analysis. TaqMan gene expression assays (Applied Biosystems, Foster City, CA, USA) were used for the following genes: *Disintegrin and metalloproteinase 10* (*Adam10*, Mm00545742_m1), *β-secretase* (*Bace1*, Mm00478664_m1), *Insulin-degrading enzyme* (*Ide*, Mm00473077_m1), and *Neprilysin* (also known as *Membrane metallo-endopeptidase Mme*, Mm00485028_m1), following the manufacturer’s instruction. Each PCR run included duplicates of reverse transcription for each sample and negative controls (reverse transcription-free samples, RNA-free sample). Quantification of the target gene transcripts was performed using *hypoxanthine phosphoribosyltransferase 1* (*Hprt1*, Mm00446968_m1) gene expression as reference, and was carried out with the 2^−ΔΔCT^ method [33].

### 2.7. Statistical Analysis

Results are presented as means ± SEM. A test was performed to detect outliers (Grubb test) and check the homogeneity of variance (Levene’s test) and data normality (Shapiro–Wilk test) for all groups. To compare the different groups, one-way ANOVA followed by the Fisher’s post hoc test was used when data were normally distributed; otherwise, the non-parametric Kruskal–Wallis test was carried out. When normally distributed data showed no sphericity, we applied an ANOVA with the Geisser–Greenhouse correction. All data were analyzed using GraphPad Prism^®^ software Version 8 (GraphPad Software Inc.). Differences in tests were considered statistically significant when *p* < 0.05.

## 3. Results

### 3.1. Amyloid Pathology

**Figure 1:**
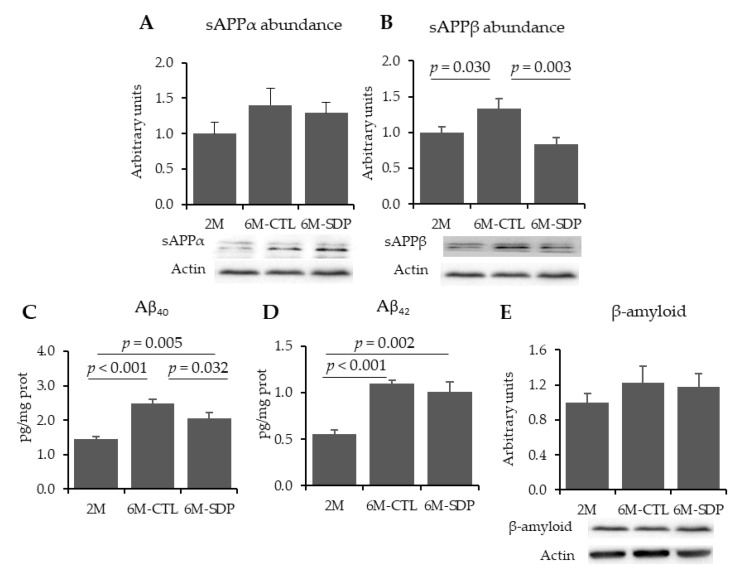
Effects of SDP supplementation on amyloid pathology markers. Panels (**A**–**E**) show the abundance of sAPPα, sAPPβ, Aβ_40,_ Aβ_42_, and β-amyloid in brain tissue. Results are expressed as mean ± SEM (*n* = 5–6 mice). Statistics: ANOVA (Fisher’s multiple comparison test). Aβ: β-amyloid; APP: amyloid protein precursor.

Neither aging nor SDP supplementation modified sAPPα abundance (Figure 1A). Aging increased the abundance of sAPPβ in the brain tissue compared to young mice (*p* = 0.030; Figure 1B). SDP supplementation reduced the abundance of this protein (*p* = 0.003). Levels of Aβ_40_ and Aβ_42_ were augmented in the cortex of 6-month-old mice (both, *p* < 0.001, Figure 1C,D, respectively), and SDP supplementation prevented Aβ_40_ increase (*p* = 0.032). Relative abundance of β-amyloid (Aβ) was not altered by senescence nor by SDP supplementation (Figure 1E).

**Figure 2:**
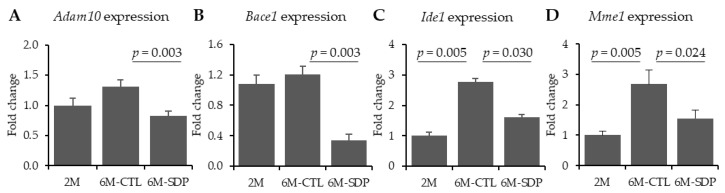
Effects of SDP supplementation on gene expression of enzymes related to aberrant APP processing. Panels (**A**–**D**) show the expression of *Adam10*, *Bace1*, *Ide1*, and *Mme1*, respectively, in cortex tissue. Results are expressed as mean ± SEM (*n* = 5–6 mice). Statistics: ANOVA (Fisher’s multiple comparison test). *Adam10*: disintegrin and metalloproteinase domain-containing protein 10; *Bace1*: *beta-secretase 1*; *Ide1*: *insulin-degrading enzyme*; *Mme1*: *neprilysin*.

Aging did not modify *Adam10* and *Bace1* gene expression (Figure 2A,B, respectively). Both genes were decreased in mice that were fed the SDP-supplemented diet (*p* = 0.003 for both enzymes). Gene expression of the Aβ degrading enzymes, *Ide1* and *Mme1*, was higher in the cortex of 6-month-old SAMP8 mice than in young mice (both *p* = 0.005, Figure 2C,D), and these effects were prevented by SDP supplementation (*p* = 0.030 and *p* = 0.024, respectively).

### 3.2. Neurofibrillary Tangles

**Figure 3:**
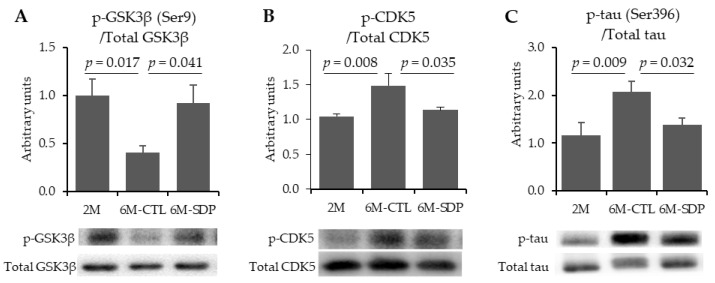
Effect of SDP supplementation on neurofibrillary tangles. Panels (**A**–**C**) show the abundance of p-GSK3β (Ser9), p-CDK5, and p-tau (Ser396) in the brain tissue, respectively. Results are expressed as mean ± SEM (*n* = 5–6 mice). Statistics: one-way ANOVA followed by Fisher’s multiple comparison test. GSK3β: glycogen synthase kinase 3β; CDK5: cyclin dependent kinase 5.

The total abundance of GSK3β and CDK5 proteins was not altered by aging nor by SDP supplementation. Nevertheless, the abundance of phosphorylated GSK3β was reduced in aged mice compared with the younger mice (*p* = 0.017, Figure 3A), and dietary supplementation with SDP prevented this change (*p* = 0.041). Moreover, aging augmented the abundance of phosphorylated CDK5 (p-CDK5; *p* = 0.008, Figure 3B), and this increase was completely prevented by SDP supplementation (*p* = 0.035). Relative abundance of total tau was not changed, neither by aging nor by SDP supplementation. However, 6M-CTL mice showed increased abundance of phosphorylated tau on Ser196 (p-tau (Ser396); *p* = 0.009; Figure 3C), and SDP supplementation prevented this increase (*p* = 0.032).

### 3.3. Neuroinflammatory Markers

**Figure 4:**
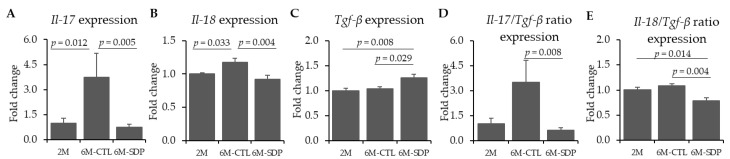
Effect of SDP supplementation on cytokine gene expression. Panels (**A**–**C**) show the gene expression of *Il-17*, *Il-18*, and *Tgf-β* in cortex tissue, respectively. Panels (**D**,**E**) show the gene expression ratio *Il-*17/*Tgf-β* and *Il-*18/*Tgf-β* in cortex tissue, respectively. Results are expressed as mean ± SEM (*n* = 5–6 mice). Statistics: one-way ANOVA followed by Fisher’s multiple comparison test. *Il:* interleukin; *Tgf-β:* transforming growth factor beta.

The gene expression of the pro-inflammatory cytokines *Il-17* and *IL-18* was increased in 6M-CTL mice compared to 2M mice (*p* = 0.012 and *p* = 0.033, respectively, Figure 4A,B), whereas the SDP supplementation prevented it (*p* = 0.005 and *p* = 0.004, respectively). Furthermore, 6M-CTL mice showed a similar gene expression of the anti-inflammatory cytokines *Tgf-β* in the cortex compared to the 2M group, while SDP supplementation increased the gene expression of this anti-inflammatory cytokine in respect to 6M-CTL mice (*p* = 0.029) and young mice (*p* = 0.008, Figure 4C). SDP supplementation reduced the ratio *Il-17*/*Tgf-β* compared to 6M-CTL mice (*p* = 0.008, Figure 4D) and the ratio of *Il-18*/*Tgf-β* in respect to senescent control mice (*p* = 0.004, Figure 4E) and young mice (*p* = 0.014).

### 3.4. Microglia Markers

**Figure 5:**
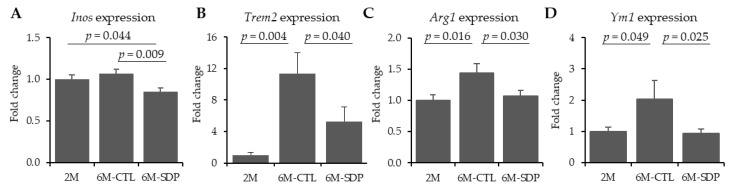
Effect of SDP supplementation on markers of microglia. Panels (**A**–**D**) show the gene expression of *Inos*, *Trem2*, *Arg1*, and *Ym1* in cortex tissue, respectively. Results are expressed as mean ± SEM (*n* = 5–6 mice). Statistics: one-way ANOVA followed by Fisher’s multiple comparison test. *Inos:* inducible nitric oxide synthase; *Trem2:* triggering receptor expressed on myeloid cells 2; *Arg1:* arginase 1; *Ym1:* chitinase-like protein 3 (Chil3).

Gene expression of *Inos* was not altered by aging (Figure 5A), but SDP supplementation diminished it (*p* = 0.009). Gene expression of *Trem2* in cortex of 6M-CTL mice was higher than in young mice (*p* = 0.004 Figure 5B), and SDP supplementation prevented it (*p* = 0.040). Moreover, the gene expression of *Arg1* and *Ym1* was also increased in aged mice (*p* = 0.016 and *p* = 0.049, Figure 5C,D, respectively), and SDP supplementation reduced these effects (*p* = 0.030 and *p* = 0.025, respectively).

## 4. Discussion

The recent approval of aducanumab, albeit with weak scientific evidence, has created a new drug for AD, as well as an anti-amyloid therapy [34]. However, no pharmacological therapy can effectively halt the decline in cognitive function associated with AD [1]. Therefore, there is growing interest in the study of protective measures to reduce disease incidence. Recent work shows that antioxidants and anti-inflammatory supplements may help decrease AD risk [35,36]. Accordingly, in this study, we examined the effects of dietary supplementation with SDP, a supplement with well-known anti-inflammatory properties. Previous studies have shown that SDP supplementation improves short- and long-term memory and increases synaptic integrity. These effects have been associated with reduced pro-inflammatory cytokine expression and increased IL-10 abundance in brain tissue [24]. Our current results show that SDP can reduce the main hallmarks of AD, such as aberrant APP processing and NFTs formation, which are substantial contributors to the disease [37].

As shown here, 6-month-old SAMP8 mice exhibited an increased amyloid and tau pathologies, as evaluated by several markers such as sAPPβ, Aβ_40_, Aβ_42_, and *p*-tau, indicating that they already had early molecular and clinical features of AD. These early events are consistent with cognitive impairment because 6-month-old SAMP8 mice already have both short- and long-term memory loss, as indicated by previous work conducted in our laboratory [24]. Activation of the amyloidogenic pathway of APP processing results in the production of sAPPβ and the generation of neurotoxic Aβ. Activation of this pathway involves the sequential cleavage of APP by β-secretase (BACE1) and γ-secretase, which results in the production of sAPPβ and the generation of neurotoxic Aβ peptides [38]. Furthermore, cleavage of APP by BACE1 is an essential first step in the production of Aβ that has been well recognized as an initial causal event of the disease [39]. Our results suggest that SDP supplementation reduces the levels of sAPPβ, as well as Aβ_40_ abundance, by decreasing the level of the amyloidogenic secretase BACE1, suggesting a reduction in the amyloidogenic pathway. These effects might explain the improved memory retention, in both the short- and the long-term, previously observed in 6-month-old SAMP8 mice supplemented with SDP [24]. Moreover, this effect of SDP is comparable to that of other nutritional interventions, which include supplementation with vitamin D, resveratrol, and lycopene, compounds, that also reduce the levels of Aβ-related biomarkers, such as APP, sAPPβ, and BACE1, in both mice and patients with AD [40,41,42].

Hyperphosphorylated tau is a neuropathological hallmark of AD that disrupts axonal transport and leads to NFTs accumulation [4]. In addition, tau phosphorylation is modulated by several protein kinases and phosphatases, such as GSK3β and CDK5 [43]. Of these kinases, CDK5 is particularly interesting because it may play a crucial role in the pathogenesis of the disease by potentially regulating the activity of other critical kinases, including GSK3β [6]. In addition, CDK5 and its potent activator p25 have been linked to the reduced synaptic density observed in early stages of AD [44]. In our hands, SDP reduced the abundance of *p*-tau and p-CDK5 while increasing that of p-GSK3β. Unlike CDK5, GSK3β is inactivated when phosphorylated in Ser9 [5]. Thus, GSK3β phosphorylation in Ser9 improves cell function and reduces NFTs levels [45]. Moreover, the effect of SDP associated with reduced abundance of activated CDK5 and p-tau is consistent with previous results showing that 6-month-old SAMP8 mice supplemented with SDP show a higher abundance of synaptophysin [24], which is indicative of higher synaptic density [46].

Although the two cardinal pathological features of AD and neuroinflammation were traditionally considered independent pathways, recent studies show an interaction between these processes, indicating that neuroinflammation might significantly affect the onset of AD [47]. Several studies indicate increased levels of pro-inflammatory cytokines in AD [48,49]. Moreover, pro-inflammatory cytokines and NF-κB signaling upregulate the BACE1 pathway, resulting in elevated Aβ production [50], and this correlates with the NF-κB increase found in aged SAMP8 mice [24]. For instance, IL-17 can activate glial cells [51], and IL-18 upregulates components of the γ-secretase complex, accelerating Aβ production, as well as GSK3β and CDK5 kinases, which promote the hyperphosphorylation of tau [52]. Moreover, reductions in microglial activation and neuronal loss are seen in IL-18 knockout mice [53]. In this study, we observed an increase in the gene expression of pro-inflammatory cytokines such as *Il-17* (and to a lesser degree *Il-18)* with age, and SDP supplementation not only prevented this effect but also increased the expression of the anti-inflammatory factor *Tgf-β*. Consequently, SDP supplementation promoted the anti-inflammatory pathway, as shown by the reduction in the *Il-17*/*Tgf-β* and *Il-18*/*Tgf-β* ratios. This might be associated with the neuroprotective effects of SDP found in the aberrant amyloid processing pathway and in NFTs. These anti-inflammatory effects of SDP correlate well with previous results showing that the SDP diet reduced the levels of NF-κB and pro-inflammatory cytokines, such as IL-6, IL-1β and TNF-α, and increased IL-10 in 6-month-old SAMP8 mice [24]. Similarly, recent studies have reported that anti-inflammatory drugs play a significant role in the AD reduction strategy [54,55].

Activated microglial cells surrounding amyloid plaques and the presence of T-cells promote neuroinflammation in AD. Indeed, there is growing interest in analyzing the role of microglia in AD because activated microglia are another key hallmark of the pathology [9]. Activated microglia are localized around senile plaques [56] and NFTs in the brain tissue of AD patients and transgenic mouse models [57]. In our hands, 6-month-old SAMP8 mice have increased hallmarks of AD and neuroinflammation, which might elevate the levels of activated M2-type microglia. Indeed, recent studies confirm that, in early stage AD, M2 phenotype microglia are activated to prevent the overproduction of Aβ and further pathological damage [58,59]. Specifically, TREM2, which is dominantly expressed by microglia in the central nervous system, is also increased in 7-month-old SAMP8 mice from [60]. Moreover, Arg1^+^ microglia have been observed to increase during IL-1β neuroinflammation in a negative feedback mechanism [61], and this cytokine is increased in 6M-SAMP8 mice, as shown in previous work [24]. These facts correlate well with our findings of increased expression of the M2 microglial markers *Trem2*, *Arg1*, and *Ym1* in aged mice. In a similar manner to our results, previous studies have shown that SDP supplementation reduces *Inos* gene expression in a mouse model of acute lung injury [21], and other natural products such as curcumin can inhibit this gene expression in microglia [62]. This relationship between neuroinflammation and activated microglia might explain the reduction in microglia markers by SDP supplementation, as SDP supplementation reduces both neuronal and systemic inflammation [19,20].

SDP is a complex mixture of functional proteins (albumin, transferrin, immunoglobulins, and other globulins), growth factors, bioactive peptides, and other biological components. Its mechanism of action may involve several of its functional components, rather than just one, which may act directly on the intestinal immune response [18], or it may promote the growth of a microbiota with anti-inflammatory effects, as demonstrated by Moretó et al. [23]. Recently, plasma protein-derived supplements have been observed to inhibit TNF secretion in rat T lymphocytes and monocytes in vitro, and to regulate the immune response of intestinal enterocytes through MyD88 and NFkB [63]. Stimulation of MyD88 and NF-κB-related receptors on enterocytes involves the activation of immunomodulatory complexes, including not only cytokine/chemokine secretion [64] but also the secretion of antibacterial peptides [65]. Conversely, there is growing evidence regarding the relationship between the gut microbiota, the digestive system, and the brain, as well as their association with inflammation [66,67,68]. Changes in the composition of the gut microbiota coextensive with aging likely contribute to immunosenescence and to the development of a pro-inflammatory phenotype [69]. In fact, SAMP8 mice at 6 months of age, showed a reduction in probiotic species, while the proinflammatory species of the intestinal microbiota increased [70]; these are able to secrete bacterial amyloids and lipopolysaccharides that are considered neurotoxic [71]. Moreover, SDP increases the presence of bacterial families that enhance the intestinal barrier function, as well as the growth of species that are well-known mediators of anti-inflammatory and tolerogenic responses [23,72]. These results suggest that some components in SDP may be effective in changing the profile of the microbiota, with potential implications for reducing intestinal and peripheral inflammation [19], and eventually communicating with to distant areas such as the brain [73].

## 5. Conclusions

**Figure 6:**
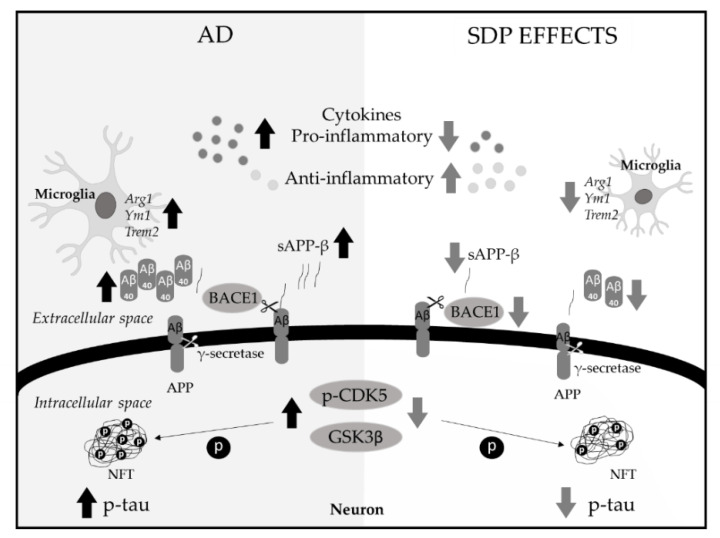
Representative scheme of the effects of SDP on AD hallmarks. SDP supplementation decreases the aberrant APP processing pathway and the hyperphosphorylation of tau. These preventive effects are accompanied by a reduction in the neuroinflammation and an attenuation in the activated microglia. APP: amyloid precursor protein, Arg1: arginase1, Aβ_40_: β-Amyloid 1-40, BACE1: β-secretase, CDK5: cyclin-dependent kinase 5, GSK3β: glycogen synthase kinase 3β, NFT: neurofibrillary tangles, P: phosphorylation, Trem2: triggering receptor expressed on myeloid cells 2, Ym1: chitinase-like protein 3 (Chil3).

In conclusion, SDP supplementation increases brain resilience against the key features of AD in SAMP8 mice, reducing the neuroinflammation and attenuating the microglial activation (Figure 6).

## Figures and Tables

**Table 1:** The composition of experimental diets.

Ingredients	Control Diet	SDP ^1^ Diet
	g/kg	
SDP	-	80
Dried skim milk	530.7	340.5
Corn starch	199.3	308.8
Sucrose	94.5	94.5
Soybean oil	70	70
Cellulose	50	50
AIN-93-G-MX (94046) ^2^	35	35
AIN-93 VX (94047) ^2^	15	15
Choline bitartrate	3	3
Methionine	2.5	3.2

^1^ SDP (spray-dried plasma) was provided by APC-Europe (Granollers, Spain). ^2^ AIN-93 MX, mineral mix AIN-93 VX, vitamin mix; both were provided by Envigo (Breso, Italy).

**Table 2:** Primers used for real-time PCR.

Primer	Forward (5′–3′)	Reverse (5′–3′)	Size (bp)
*Arg1*	GACAGGGCTCCTTTCAGGAC	GCCAAGGTTAAAGCCACTGC	108
*Il-17*	AGCAGCGATCATCCCTCAA	TTGCGCCAAGGGAGTTAAA	104
*Il-18*	GTTTACAAGCATCCAGGCACAG	GAAGGTTTGAGGCGGCTTTC	151
*Inos*	CCCAACAATACAAGATGACCCT	AGGGATTCTGGAACATTCTGTG	87
*Tgf-b*	CAGGGTGAAGGGGAAAACTC	AGTTCGGTCATTAGTCTCGC	204
*Trem2*	CCTGAAGAAGCGGAATGGGA	CTTGATTCCTGGAGGTGCTGT	269
*Ym1*	GTGGAGAAAGACATTCCAAGGC	CAGTTCAGGGATCTTGTACCCA	90

*Arg1:* arginase1; *Il:* interleukin; *Inos: inducible nitric oxide synthase*; *Tgf-b:* transforming growth factor beta; *Trem-2:* triggering receptor expressed on myeloid cells 2; *Ym1:* chitinase-like protein 3 (Chil3).

## Data Availability

The datasets generated during and/or analyzed during the current study are available from the corresponding author on reasonable request.
